# Cross-sectional survey on researchers’ experience in using accelerometers in health-related studies

**DOI:** 10.1136/bmjsem-2021-001286

**Published:** 2022-05-09

**Authors:** Birte Marie Albrecht, Fabian Tristan Flaßkamp, Annemarie Koster, Bjoern M Eskofier, Karin Bammann

**Affiliations:** 1 Institute for Public Health and Nursing Research (IPP), University of Bremen, Bremen, Germany; 2 Leibniz ScienceCampus Digital Public Health, Bremen, Germany; 3 Department of Social Medicine, CAPHRI, Care and Public Health Research Institute, Maastricht University, Maastricht, The Netherlands; 4 Department Artificial Intelligence in Biomedical Engineering, Friedrich-Alexander University Erlangen-Nuernberg, Erlangen, Germany

**Keywords:** accelerometer, epidemiology, measurement, physical activity, research

## Abstract

**Objectives:**

Accelerometers are widely applied in health studies, but lack of standardisation regarding device placement, sampling and data processing hampers comparability between studies. The objectives of this study were to assess how accelerometers are applied in health-related research and problems with accelerometer hardware and software encountered by researchers.

**Methods:**

Researchers applying accelerometry in a health context were invited to a cross-sectional web-based survey (August 2020–September 2020). The questionnaire included quantitative questions regarding the application of accelerometers and qualitative questions on encountered hardware and software problems. Descriptive statistics were calculated for quantitative data and content analysis was applied to qualitative data.

**Results:**

In total, 116 health researchers were included in the study (response: 13.7%). The most used brand was ActiGraph (67.2%). Independently of brand, the main reason for choosing a device was that it was the standard in the field (57.1%–83.3%). In children and adolescent populations, sampling frequency was higher (mean: 73.3 Hz ±29.9 Hz vs 47.6 Hz ±29.4 Hz) and epoch length (15.0s±15.6s vs 30.1s±25.9s) and non-wear time (42.9 min ±23.7 min vs 65.3 min ±35.4 min) were shorter compared with adult populations. Content analysis revealed eight categories of hardware problems (battery problems, compliance issues, data loss, mechanical problems, electronic problems, sensor problems, lacking waterproofness, other problems) and five categories of software problems (lack of user-friendliness, limited possibilities, bugs, high computational burden, black box character).

**Conclusions:**

The study confirms heterogeneity regarding accelerometer use in health-related research. Moreover, several hardware and software problems were documented. Both aspects must be tackled to increase validity, practicability and comparability of research.

**Key messages**

**What is already known**

- Accelerometers are increasingly used for objectively measuring physical activity in health-related studies but to this date, no real standard has evolved for collection, preprocessin and analyses of accelerometer data in health-related studies.

**What are the new findings**

- A wide range of methods in the application of accelerometers were observed which can partially be attributed to research objectives and study population.
- Participating researchers were not satisfied with the currently available software and there is a need for easy-to-use software solutions that are well documented, flexible and bug-free.
- Researchers applying accelerometry in health-related studies are facing several hardware problems, which underlines the potential for improvement of accelerometer devices.

**How this study might affect research, practice or policy**

- Efforts to harmonise methods for accelerometer data collection and processing are needed and reported problems with accelerometer software and hardware must be tackled.

## Background

Insufficient physical activity (PA) is one of the major determinants of mortality worldwide^1^ with around three million premature deaths in 2009.^2^ Sufficient PA is essential for optimal body functioning^2^ and reduces the risk of most chronic diseases.^3^ In addition to its value in primary prevention, PA also has a relevant role in the therapy of several diseases including psychiatric, neurological, metabolic, cardiovascular and musculoskeletal diseases as well as cancer.^4^ This illustrates the importance of large epidemiological studies on PA which require a valid, practical and acceptable method for measuring PA.

Since the mid-90s, the use of accelerometry for the measurement of PA has increasingly become popular as an alternative to self-reported questionnaires.^5 6^ For accelerometry, study participants wear a small device (‘accelerometer’) at the body, commonly either at the hip, wrist, thigh or ankle over a specific time period (usually several days) that measures accelerations and decelerations of the body in one to three axes. Historically, the collected raw accelerations were converted into unit less counts before applying metrics like, for example, vector magnitudes to the accelerometer data. However, this conversion, which is based on closed, proprietary algorithms of the accelerometer manufacturers, is criticised and approaches avoiding this step, for example, Euclidian Norm Minus One gravity, are becoming more and more common.^6^ Subgroup-specific cut-points can be applied to the acceleration data to estimate PA intensity levels,^7 8^ which enables to evaluate whether PA recommendations are met. Accelerometry is not only applied for the measurement of PA, but also to estimate parameters of sedentary time,^9^ sleep^10^ or to identify specific activities.^11 12^

Nowadays, accelerometry is commonly applied in health research. Yet, there is still a lack of standardisation in all parts of the process, even if identical devices are used and similar populations studied. This hampers comparability between these studies.^13^ A review by Welk *et al* documents the heterogeneous nature of methods researchers employ for analysing accelerometer data and reporting of results.^14^ However, large heterogeneity exists also regarding accelerometer brands, accelerometer placement, sampling options and data preprocessing.^15^

To further investigate this heterogeneity, we conducted a cross-sectional survey among health researchers who applied accelerometry. The aim of this study was to assess (1) how accelerometers are used in health-related research including information on data collection, preprocessing and analyses and (2) what problems are encountered by the researchers regarding hardware and software.

## Methods

### Study population

We conducted a web-based cross-sectional questionnaire between August and September 2020 via the SoSci Survey online tool (SoSci Survey, Munich, Germany). For the recruitment of participants: (1) authors of publications published between January 2018 and August 2020 under the MeSH term ‘accelerometry’ in MEDLINE accessed through PubMed (https://pubmed.ncbi.nlm.nih.gov/) and (2) contact persons of registered clinical trials under the search term ‘accelerometry’ in the US National Library of Medicine (https://www.clinicaltrials.gov/) and the European Clinical Trials Register (https://www.clinicaltrialsregister.eu/) were identified. All results were screened manually by one author to ensure that the project was relevant for the research question. If the contact details were not provided on PubMed or the clinical trial register, the contact details were extracted from the respective institutional websites. Researchers were only contacted if they were still associated with the institution.

The invitation via email briefly explained the purpose of the study and contained an embedded link to the questionnaire. Due to the anonymity of the questionnaire, a reminder was sent to all potentially eligible individuals in the beginning of September. Only participants who had entered their contact details to receive information on the study results at the end of the questionnaire did not receive a reminder.

All participants provided informed consent at the beginning of the questionnaire.

### Patient and public involvement

Patients and/or the public were not involved in the design, or conduct, or reportin or dissemination plans of this research.

### Measures

All data were assessed via self-administered web-based questionnaire (see online supplemental appendix 1). The questionnaire was self-developed and feedback was sought by experts. Multiple answers were allowed for questions regarding experience with accelerometer data, studied population (children and adolescents (0–17 years), adults (18–64 years), older adults (≥65 years)), measurement purpose, accelerometer brand, accelerometer placement, software used and reasons for choosing the device. Additionally, participants were able to provide further information in a text box. Open-ended questions were used for the assessment of sampling frequency (in Hz), minimum sampling days and hours per day, non-wear time (in minutes) and epoch length (in seconds). Qualitative data on major problems encountered (1) caused by the monitoring device and (2) working with software for downloading and processing accelerometer data were also collected via open-ended questions.

10.1136/bmjsem-2021-001286.supp1 Supplementary data

### Analyses of quantitative and qualitative data

Absolute and relative frequencies for quantitative data on researchers’ experience with accelerometer data, studied populations, purpose of measurement, accelerometer placement, accelerometer brand and software used were calculated for the total population. Absolute and relative frequencies for reasons for choosing the device were analysed stratified by the device used. Means and SD were determined for sampling frequency (in Hz), minimum sampling days, minimum sampling hours per day, non-wear time (in minutes) and epoch length (in seconds) for the total population as well as for research on children/adolescents and adults/older adults, respectively. In addition, absolute and relative frequencies were calculated for the most stated answers. If data was missing for specific variables, participants were not included for that part of the analyses. All analyses of quantitative data were performed in SPSS Statistics V.22.0 (IBM).

Qualitative data obtained from the open questions on problems encountered while using hardware and software were transcribed. Content analysis was applied and the answers were coded on the basis of similarity in meaning by one of the authors. Derived categories were then discussed with a second author.

## Results

In total, 862 individuals were invited to participate in the web-based questionnaire via e-mail in August 2020 (table 1). Most invited researchers were either professors (34.9%) or had a doctoral title (45.7%) and were based in Europe (54.1%). Five researchers responded that they themselves were unable to participate and three of those subsequently forwarded the invitation to an eligible colleague. The questionnaire was accessed 396 times, this includes instances in which it was accessed multiple times by a single person. A total of 140 individuals started the questionnaire, of these, 22 aborted midway. A total of 118 researchers completed the survey (response: 13.7%). Of these, 2 researchers reported pedometer use only, leaving 116 researchers that were included in the analyses.

**Table 1:** Description of invited scientists, n (%)

	n=862
Academic title	
Professor	301 (34.9)
PhD	394 (45.7)
Master’s degree or lower	167 (19.4)
Continent	
Africa	1 (0.1)
Australia	67 (7.8)
Asia	20 (2.3)
Europe	466 (54.1)
Middle/South America	19 (2.2)
North America	289 (33.5)

Most researchers had experience with analysis and interpretation of accelerometer data, and often also with pre-processing (70.7%; table 2). The accelerometer was predominantly placed at the hip (59.5%) and wrist (46.6%), thigh (18.1%) and ankle (9.5%) placement were less common. The most used accelerometer brand was ActiGraph (67.2%), followed by activPAL (15.5%), Axivity (12.1%), GENEActiv (12.1%) and SenseWear (6.0%). The category ‘other devices’ included 20 different accelerometer brands, which were mentioned by 26 participants (22.4%). Most of the participating researchers used shelf software (73.2%), including ActiLife (62.2%) and the R package GGIR (20.7%). Own software implementations were used by 26.8%.

**Table 2:** Description of the responding researchers, n (%)

	n=116
Experience with accelerometer data	
Interpretation only	21 (18.1)
Interpretation and data preprocessing	13 (11.2)
Interpretation, (data preprocessing) and data analysis	82 (70.7)
Studied populations	
Only children and adolescents (0–17 years)	28 (24.8)
Only adults (18–64 years)	16 (14.2)
Only older adults (≥65 years)	15 (13.3)
Children and adolescents (0–17 years)+adults (18–64 years)	13 (11.5)
Children and adolescents (0–17 years)+older adults (≥65 years)	3 (2.7)
Adults (18–64 years)+older adults (≥65 years)	23 (20.4)
All three age groups	15 (13.3)
Purpose of measurement	
Outcome only	69 (59.5)
Exposure or confounder only	12 (10.3)
Outcome and exposure/confounder	31 (26.7)
Other	4 (3.4)
Accelerometer placement (multiple answers)	
Hip	69 (59.5)
Wrist	54 (46.6)
Thigh	21 (18.1)
Ankle	11 (9.5)
Other placements	17 (14.7)
Accelerometer brand (multiple answers)	
ActiGraph	78 (67.2)
activPAL	18 (15.5)
Axivity	14 (12.1)
GENEActiv	14 (12.1)
SenseWear	7 (6.0)
Other devices	26 (22.4)
Software used	
Shelf software	82 (73.2%)
Of these (multiple answers)…	
…ActiLife	51 (62.2%)
…R package GGIR	17 (20.7%)
Own software implementations	30 (26.8%)

Table 3 shows the reasons for choosing the device stratified by the accelerometer brand used. The main reasons for choosing a device—independent of the accelerometer brand—were that it was the standard device in the field and for comparability with other studies. Only for ‘other devices’ the main reason were technical specifications (57.7%).

**Table 3:** Reasons for choosing the device (multiple answers) stratified by used accelerometer brand, n (%)

	ActiGraph n=78	activPAL n=18	Axivity n=14	GENEActiv n=14	SenseWear n=7	Other device n=26
Reasons						
Standard device in the field	64 (82.1)	15 (83.3)	8 (57.1)	9 (64.3)	5 (71.4)	12 (46.2)
Comparability with other studies	58 (74.4)	10 (55.6)	8 (57.1)	10 (71.4)	4 (57.1)	12 (46.2)
Technical specifications	31 (39.7)	7 (38.9)	6 (42.9)	7 (50.0)	3 (42.9)	15 (57.7)
Provides unique features	14 (17.9)	7 (38.9)	2 (14.3)	5 (35.7)	2 (28.6)	11 (42.3)
Manufacturer provides software	17 (21.8)	3 (16.7)	2 (14.3)	4 (28.6)	3 (42.9)	7 (26.9)
High compliance	16 (20.5)	7 (38.9)	6 (42.9)	7 (50.0)	1 (14.3)	4 (15.4)

Settings of variables for data collection and preprocessing are described in table 4. Means as well as absolute and relative frequencies of the most commonly used settings are displayed for all study populations in total and separate for researchers that exclusively have experience in one of the two populations (children/adolescents or adults/older adults). Mean sampling frequency was 61.2 Hz ±31.9 Hz in total and was higher among children/ adolescents (73.3 Hz ±29.0 Hz) compared with adults/older adults (47.6 Hz ±29.4 Hz). The mean of minimum sampling days was 3.8±1.6 with most researchers using three (21.7%) or 4 days (48.9%). The results were similar for both age groups. Mean minimum sampling hours per day was 10.4±4.0. A minimum of eight sampling hours per day was more common in the younger age group (children/adolescents: 36.4% vs adults/older adults: 6.1%) while a minimum of ten sampling hours was more often used in the older age group (adults/older adults: 66.7% vs children/adolescents: 31.8%). Mean definition of non-wear time was lower for children/adolescents (42.9 min ±23.7 min) compared with adults/older adults (65.3 min ±35.4 min). However, in both age groups non-wear time was most often defined as 60 min (children/adolescents: 47.1% vs adults/older adults: 42.9%). The mean of predominantly used epoch length was 22.7 s±23.1 s in total, 15.0 s±15.6 s in children/adolescents, and 30.1 s±25.9 s in adults/older adults. In children/adolescents, epoch length was most often set at 15 s (50.0%) and in adults/older adults at 60 s (40.0%). The most frequently reported format for accelerometer data were activity counts (65.5%), followed by raw acceleration (42.2%) and step counts (37.9%).

**Table 4:** Variables of data collection and pre-processing

	Total	Researchers studying exclusively children or adolescents	Researchers studying exclusively (older) adults
	n=116	n=28	n=54
Sampling frequency (in Hz)	Mean (SD) 61.2 (31.9)	Mean (SD) 73.3 (29.0)	Mean (SD) 47.6 (29.4)
	Range 10–100	Range 30–100	Range 10–100
Of these*	n (%)	n (%)	n (%)
30 Hz	23 (28.0)	4 (19.0)	12 (33.3)
100 Hz	27 (32.9)	9 (42.9)	6 (16.7)
Minimum sampling days	Mean (SD) 3.8 (1.6)	Mean (SD) 4.0 (2.2)	Mean (SD) 3.8 (1.3)
	Range 1–12	Range 1–12	Range 1–7
Of these*	n (%)	n (%)	n (%)
3 days	20 (21.7)	7 (26.9)	6 (15.4)
4 days	45 (48.9)	10 (38.5)	20 (37.0)
Minimum sampling hours per day	Mean (SD) 10.4 (4.0)	Mean (SD) 9.3 (3.1)	Mean (SD) 10.8 (4.4)
	Range 1–24	Range 5–16	Range 1–24
Of these*	n (%)	n (%)	n (%)
8 hours	14 (18.2)	8 (36.4)	2 (6.1)
10 hours	42 (54.5)	7 (31.8)	22 (66.7)
Non-wear time (in minutes)	Mean (SD) 59.2 (32.7)	Mean (SD) 42.9 (23.7)	Mean (SD) 65.3 (35.4)
	Range 1–150	Range 10–90	Range 1–150
Of these*	n (%)	n (%)	n (%)
60 min	29 (44.6)	8 (47.1)	12 (42.9)
90 min	15 (23.1)	1 (5.9)	9 (32.1)
Epoch length (in s)	Mean (SD) 22.7 (23.1)	Mean (SD) 15.0 (15.6)	Mean (SD) 30.1 (25.9)
	Range 1–60	Range 1–60	Range 1–60
Of these*	n (%)	n (%)	n (%)
1 s	16 (17.8)	5 (20.8)	6 (15.0)
15 s	17 (18.9)	12 (50.0)	1 (2.5)
60 s	23 (25.6)	2 (8.3)	16 (40.0)
Accelerometer data format	n (%)	n (%)	n (%)
Activity counts	76 (65.5)	16 (57.1)	32 (59.3)
Raw acceleration	49 (42.2)	15 (53.6)	17 (31.5)
Step counts	44 (37.9)	3 (10.7)	25 (46.3)
MET minutes	26 (22.4)	2 (7.1)	13 (24.1)
ENMO	19 (16.4)	6 (21.4)	6 (11.1)
Energy expenditure (in kJ/kcal)	13 (11.2)	1 (3.6)	6 (11.1)
Mean amplitude	10 (8.6)	2 (7.1)	1 (1.9)

*Most prevalent options.

ENMO, Euclidian Norm Minus One; MET, metabolic equivalent.

Derived categories and findings resulting of the qualitative analyses regarding problems handling hardware and software are shown in table 5. Eight categories were derived for hardware problems: battery problems, compliance issues, data loss, mechanical problems, electronic problems, sensor problems, lacking waterproofness and other problems. Most frequent hardware problems were issues around the battery. Researchers referred to the loss of battery life in devices after expiration of warranty. Another problem is shorter battery time during cold weather. As one participant observed: ‘Low battery capacity in freezing temperatures [… is a] problem during winter time in studies, where the devices are mailed to participants.’ Likewise, several issues were reported that led to a lack of compliance, mostly related to wearing comfort of the devices. Other hardware issues referred to data loss, electronic and sensor problems. Mechanical problems as break of casing, caps, and wrist bands were reported, which also happened during data collection: ‘(brand name) wrist straps regularly spontaneously fall apart, meaning the device cannot continue to be worn unless a replacement strap can be provided’. Lastly, the lack of waterproofness of devices is a problem for uninterrupted data collection and complete assessment of PA.

**Table 5:** Reported problems with accelerometer hardware and software in health research

Hardware problems		Software problems	
Derived categories	Findings	Derived categories	Findings
Battery problems	Battery problems were quite frequently reported. Battery life shortens over time and during cold weather periods, resulting in problems of data collection and data loss.	Lack of user-friendliness	Some users felt that often too many steps were necessary to process the data. Packages running in the R software environment (GGIR, accelerometry) were repeatedly reported as being too complicated to use.
Compliance issues	Another frequent problem. Compliance is reported to differ by placement, and consequently by device with best compliance in wrist-worn devices. Also, skin irritation has been reported. Children are especially prone to non-wearing uncomfortable devices, and to loss of devices. Moreover, children in a classroom setting will swap devices if not labelled.	Limited possibilities of software	Only a limited number of settings is available with shelf software.
Data loss	Data loss due to technical failure was reported frequently, without giving specific causes. In some cases, the manufacturer was able to rescue the data.	Software bugs	Software bugs were reported in varying detail. No general finding.
Mechanical problems	The accelerometer casings, the wrist straps, and other mechanical components were frequently reported to break.	High computational burden	High time complexity was repeatedly reported to be a problem with the Actilife software.
Electronic problems	There were some reports on faulty internal clocks, memory and Bluetooth issues.	Black box character of software	Data processing of software is not well documented, as a result the software appears non reliable to the user. This also poses a problem for scientific reporting as exact procedures are not known.
Sensor problems	Faulty signals, filtering or calibration issues were reported infrequently.		
Lacking waterproofness	Lack of waterproofness is a problem, especially when active swimmers are in the sample. Some reported that they would manually add the activities to the derived parameters, which is cumbersome.		
Other	Lack of comparability between devices, bankruptcy of one device manufacturer. General doubts on validity of the devices.		

For software problems, five categories emerged: lack of user-friendliness, limited possibilities of software, software bugs, high computational burden and black box character of software. The most frequently mentioned problem with accelerometer software were the perceived lack of user friendliness and a lack of transparency of the software procedures, which results in a lack of control for the researchers. One researcher stated: ‘(R package) oftentimes will not run smoothly and its error messages are impossible to decipher. So you know you made a mistake somewhere in your code or in your file structure, but you have no idea where to look for your mistake.’ Another researcher observed: ‘Software can also be quite black-boxed making it difficult to report calculations or identify data errors’. A third problem were the number of bugs encountered with some of the programmes: ‘[…] we try to just fix the bugs we identify’. Consequently, some researchers reported to use own solutions: ‘Due to significant problems I encountered, I stopped working with [the programs], export the data as direct as possible and process myself’.

## Discussion

Our study displayed the wide heterogeneity in the practice of accelerometer use in health research studies and a number of problems with accelerometry use was documented. A large range of different practices were observed regarding collection and handling of data. Some of these differences can be attributed to the research objectives and the populations under study. For example, when studying children higher sampling frequency, shorter non-wear time definitions and shorter epochs compared with studies in adult population were more frequent. Nevertheless, no real standard has evolved so far, even not for specific populations or parameters.^13 16^ On top of this, the different brands of accelerometers, and the different ways of reporting results^14^ hampers comparability of published research. Recently, first endeavours towards the development of accelerometry standards have been undertaken, although separately for data collection and data analyses.^14 17–19^ Another example is the placement-specific initiative Prospective, Physical Activity, sitting, and Sleep (ProPASS) consortium on thigh-worn accelerometers,^20 21^ aiming to develop methods for processing, harmonising and pooling data of existing cohort studies and by providing methods and guidance for prospective harmonisation. Apart from enhancing comparability of research results, established standards would diminish burden from the researchers.

The study showed a need for accelerometer software that is well documented, flexible, easy-to-use and bug-free. The current situation is by no means satisfying and many researchers feel overwhelmed by the task of analysing the data with the currently available solutions. Moreover, the use of black box software or proprietary algorithms contradicts basic scientific principles as the researcher is not able to give account to the exact analytic procedures the data underwent. Accelerometer software should at best follow FAIR (Findable, Accessible, Interoperable, Reusable) principles.^22 23^

The participating researchers frequently reported hardware problems with the accelerometer devices. Most of the reported problems could principally be overcome by using higher quality construction parts and assembly. With sales figures still on the rise^5 6^ there might be not much appeal for manufacturers to invest in better hardware, even less if spare parts and attachment straps build a substantial part of their revenues. To prevent loss of battery capacity in cold weather, batteries should be protected on their way to the participants. Batteries that perform in cold weather conditions are currently being investigated.^24^

This study has limitations. Although some effort was done to gather a sampling frame that covers the majority of health researchers working currently with accelerometers, we did not include researchers that were neither publishing nor conducting a registered clinical trial in the field between January 2018 and August 2020. The response proportion was 13.8%, and it might well be that researchers experiencing problems with accelerometers were more likely to answer the questionnaire than those who did not. Moreover, considering the vast range of accelerometers hardware, software, methods to analyse and ways to include them into research is somewhat challenging for a questionnaire with a limited set of mostly closed-ended questions. While keeping the questionnaire short, the survey could only scratch on the surface of the problem.

## Conclusions

The results of our survey on the application of accelerometry in health-related research underlines the wide range of methods used in this field, which can be partially attributed to research objectives and population. Researchers are facing several problems regarding accelerometer hardware and software, which should be tackled to facilitate the application. To our knowledge, this is the first survey of this kind. It elucidates the subjective perspective of users and complements the results of systematic reviews. Many of the participating researchers expressed interest in the results, which underlines the scarcity of published information on practical problems and solutions. Although being the most favourable method for PA assessment, accelerometer research needs to tackle the practical problems that became very obvious during our research. Collaborative efforts, such as ProPASS, are important to pave the way towards harmonised methods for accelerometry data collection and processing.

## Data Availability

Data are available on reasonable request from the corresponding author.
