# Supplementary material for: Cross-sectional survey on researchers’ experience in using accelerometers in health-related studies
Source: BMJ Open Sport Exerc Med. 2022 May 9;8(2):e001286. doi: 10.1136/bmjsem-2021-001286 (PMC9086608; doi:10.1136/bmjsem-2021-001286)
Supplement: Supplementary data [file bmjsem-2021-001286supp001.pdf]

Welcome to the Survey for Activity Monitor Use in Health Research

We are a group of academic researchers from the universities of Bremen and Erlangen-Nürnberg (Germany) planning a methods project where we would like to improve usability and user-friendliness of activity monitors in health research. An expected result is open source software, and possibly a new prototype hardware solution. The project builds upon our own practical experiences with activity monitors for physical activity assessment in epidemiological studies. Although we believe in the technology, we too often encountered shortcomings and annoyances in currently available solutions.

To build a stronger base, we would like to add your experience as users of activity monitors in health research. What obstacles did you encounter when using activity monitoring in your research? How should the ideal device perform? What health-related measures are you interested in? What do you expect from the activity monitor software? Our aim is to survey how the monitoring devices are being applied to current research. The questionnaire consists of five sections. The first four sections evaluate the role and application of the monitors within your research. The fifth section contains questions about current challenges and demands for future devices and software. The survey will take about 15 minutes to complete. You can pause at any time and resume later.

You have been invited to participate because you are either listed in a clinical trial register or participated in a scientific publication on the topic. If you feel this invitation should (also) go to a different person working with data derived from activity monitors, please feel free to forward it to this person.

Your involvement in the survey is voluntary, and you may choose not to participate or stop at any time. All information is recorded and stored anonymously and cannot be traced back to individuals. The data will only be used within this research project and will neither be exploited commercially nor accessed by third parties. Your data will be handled confidentially in accordance with European data protection law (DSGVO).

For any further information, please contact *flasskamp@uni-bremen.de*

Thank you for your participation!

Sincerely,

Karin Bammann[1,2], Björn Eskofier[3] and Fabian Flaßkamp[1,2]

[1]Institute for Public Health and Nursing Research, University of Bremen
[2]Leibniz Science campus for Digital Public Health
[3]Machine Learning & Data Analytics Lab, University of Erlangen-Nürnberg

1. **Does your current project involve accelerometry?**
   - Yes
   - No

2. **In which part(s) of the activity measurements are you involved?**
   (multiple answers)
   - Planning and management
   - Fieldwork
   - Data downloading/Data preparation
   - Data analysis/statistical modeling
   - Data interpretation
   - Other: *Text*

3. **What is/are your research population(s)?**
   (multiple answers)
   - Children/youths (0-17 years old)
   - Adults (18-64 years old)
   - Older adults (65+ years old)
   - Other: *Text*

4. **What is the purpose of the accelerometer-derived data you are collecting?**
   (multiple answers)
   - Main outcome
   - Secondary outcome
   - Main exposure
   - Secondary exposure
   - Confounder
   - Other: *Text*

5. **Which activity monitor are your using for your current research?**
   (multiple answers)
   - ActiCal
   - ActiGraph GT3X family
   - ActiGraph GT1M
   - activPAL
   - IDEEA family
   - SenseWear
   - Other: *Text*
   - I don’t know

6. **Why did you choose this model?**
   (multiple answers)

   - Standard device in this field
   - Comparability with other studies
   - Provides unique measures/features
   - Technical specifications (i.e. long battery life, large storage space, etc.)
   - Manufacturer provides software
   - Has a screen
   - Has no screen
   - High compliance
   - Other: *Text*

7. **Where is/are the device(s) placed on the body?**
   You can select the appropriate marker on the right side. Point your cursor at the appropriate location on the body where the accelerometer is placed and do a single left-click. The selected marker will appear to indicate the position. You can also drag & drop the markers to adjust the location or left-click them again to make them disappear.
   If necessary, you may use the with circle and square symbols to indicate (non-)dominant sides by placing them close to the marker.

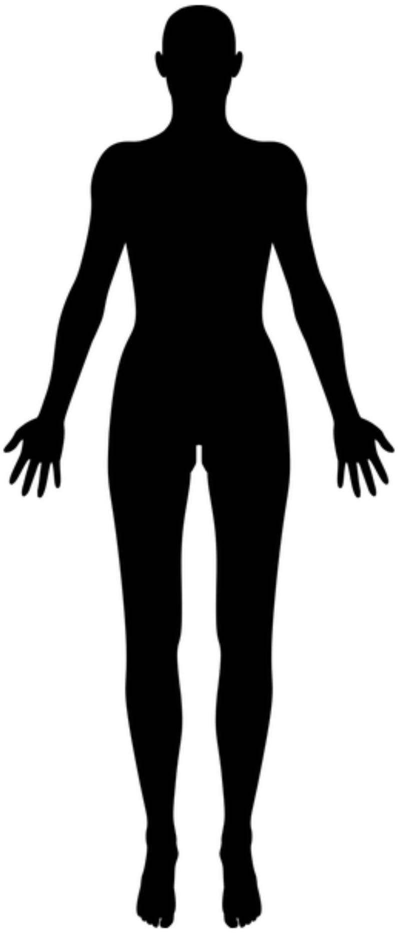

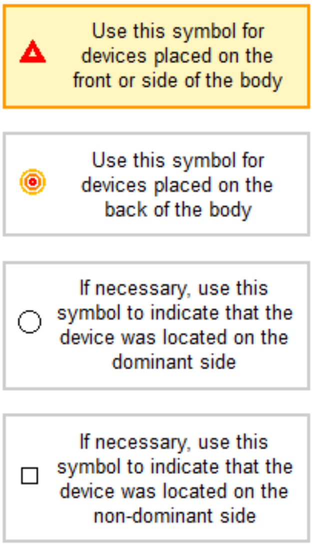

8. At what rate are you sampling?

- *Numbers* Hertz
- I don't know

9. Which program(s) are you using to download and process the accelerometer-derived data?
(multiple answers)

- ActiLife
- ActiSci
- Body Media InnerView Research Software
- Freiburg monitoring system
- KineSoft
- MAHUffe
- R-Package "GGIR"
- R-Package "Physical activity"
- SAS SUDAAN
- Other: *Text*
- I don't know

10. What is the epoch length you use for your analyses?

- Predominantly using: *Numbers* seconds
- Other definition: *Numbers*
- Not applicable

11. Please describe shortly how you defined non-wear time:

- *Numbers* minutes of continuous counts of zero activity with a tolerance of up to *numbers* minutes of activity
- Other definition: *Text*
- I don't know

12. What amount of wear-time is required for a dataset to be considered valid in your research?

- *Numbers* days with at least *numbers* hours of recorded wear-time
- Other definition: *Text*
- I don't know

13. Please specify if you ever have encountered major problems caused by the monitoring device?
By that, we mean problems that are caused by the device itself and not by, for example, accompanying software, etc.

- *Text*
- I have not encountered any major problems
- I don't know
- I have not worked with the monitoring device

Albrecht BM, *et al. BMJ Open Sp Ex Med* 2022; 8:e001286. doi: 10.1136/bmjsem-2021-001286

14. What is/are the format/s of the accelerometer-derived data you are typically using?
(multiple answers)

- o Activity counts
- o Raw acceleration
- o ENMO
- o Mean amplitude
- o Step counts
- o Energy expenditure in kJ/kcal
- o MET minutes
- o Other: *Text*
- o I don't know (yet)

15. Do you classify your data into PA levels?

- o Yes
- o No
- o I don't know

16. Which intensities are relevant for your research?
(multiple answers)

- o Sedentary time (<1,5 MET)
- o Low PA (≥1,5 MET, <3 MET)
- o Moderate PA (≥3 MET, <6 MET)
- o Vigorous PA (≥6 MET)
- o MVPA
- o Sleep time
- o Other definitions: *Text*

17. Are you using established cut-offs, or are you developing them specifically for this research?

- o Established cut-offs
- o Newly developed cut-offs
- o I don't know

18. Please specify if there is a need for any validation studies that would help your research (for example, validation of PA intensity cut-points for different subgroups/accelerometer placements, validations using a specific statistical method, etc.)?

Text

19. Below, you will find a list of features for software downloading and processing accelerometer-derived data. Please rate how important each feature is for your research.

Data downloading and exporting

	Not at all important	Moderately important	Very important	I don't know
Detection and deletion options for implausible values	○	○	○	○
Data vizualisation	○	○	○	○
Output in different file formats	○	○	○	○
Conversion of raw data to activity counts	○	○	○	○
Algorithmic estimation of non-wear time	○	○	○	○

Are there any other important features not mentioned in the list above?

Text

Calculation options

	Not at all important	Moderately important	Very important	I don't know
Single axes options	○	○	○	○
Vector magnitude options	○	○	○	○
Calculation options for hip placement	○	○	○	○
Calculation options for wrist placement	○	○	○	○
Calculation options for thigh placement	○	○	○	○
Calculation options for ankle placement	○	○	○	○
Calculation options for multiple placements	○	○	○	○
Activity recognition	○	○	○	○
Options for bouts	○	○	○	○

Are there any other important features not mentioned in the list above?

Text

Parameter outputs

	Not at all important	Moderately important	Very important	I don't know
Identification of posture (lying, standing, sitting)	○	○	○	○
MVPA analyses	○	○	○	○
Sedentary activity recognition	○	○	○	○
Sleep time (including indicators of sleep quality)	○	○	○	○
Estimation of energy expenditure	○	○	○	○
Step counts	○	○	○	○

Are there any other important features not mentioned in the list above?

Text

20. **Please specify if you have ever encountered major problems working with software meant to download and process accelerometer data?**
    - *Text*
    - I have not encountered any major problems
    - I have not worked with software meant for accelerometer data downloading and processing
    - I don't know

21. **We understand that the field of accelerometry-based research is broad. If at any point you found that the answers did not account for any of the scenarios in your research or if you have any other comments, feedback, additions, or critique, please leave a comment.**

    Text

Albrecht BM, *et al. BMJ Open Sp Ex Med* 2022; 8:e001286. doi: 10.1136/bmjsem-2021-001286
